# Immunoglobulin A Vasculitis Nephritis in an Adult

**DOI:** 10.7759/cureus.72672

**Published:** 2024-10-29

**Authors:** Sritheja Gopalakrishnan, Nirmala Devi Chandrasekaran, Janardanan Kumar, Chandni Jayakumar, Varadharajan Jayaprakash

**Affiliations:** 1 General Medicine, Sri Ramaswamy Memorial (SRM) Medical College Hospital and Research Centre, Sri Ramaswamy Memorial (SRM) Institute of Science and Technology, Chengalpattu, IND; 2 Nephrology, Sri Ramaswamy Memorial (SRM) Medical College Hospital and Research Centre, Sri Ramaswamy Memorial (SRM) Institute of Science and Technology, Chengalpattu, IND

**Keywords:** adult onset, corticosteroids, eular, iga vasculitis (igav), varicella

## Abstract

Immunoglobulin A vasculitis (IgAV; initially known as Henoch-Schönlein purpura) is a form of vasculitis involving the small blood vessels of the gastrointestinal tract, skin, joints, and kidney, presenting as a multisystem disorder. A 21-year-old gentleman presented with joint pain, skin rash, abdominal pain, and proteinuria. Biopsies performed from the skin and kidney were both consistent with IgA deposition. The patient was treated with a short course of low-dose steroids because of the involvement of multiple organ systems. His symptoms abated, proteinuria resolved, and steroids were stopped after four weeks. He remains in complete remission at the time of the last follow-up, 12 months after the initial presentation.

## Introduction

Immunoglobulin A vasculitis (IgAV; initially referred to as Henoch-Schönlein purpura) is an immune-mediated small-vessel vasculitis, which can involve multiple organ systems, namely, the gastrointestinal tract, kidneys, skin, and musculoskeletal system. This condition needs to be differentiated from IgA nephropathy, which has a similar pathogenesis but involves only the kidneys without other systemic manifestations. There are varied presentations and a vast array of triggering factors, documented to play a role in etiopathogenesis. It is usually diagnosed on clinical grounds, coupled with a biopsy obtained from lesions in the skin and/or kidneys. Currently, there are no established criteria to diagnose IgAV in adults; the criteria for the pediatric population are extrapolated to the adult population [1]. There are very few case reports in the literature, on the association of IgAV with varicella [2-5], which are predominantly in the pediatric population. Here, we discuss a case of IgAV in a man in his third decade, with a varicella infection, about three months prior to onset.

## Case presentation

A 21-year-old male, with no known comorbidities, presented to us with a history of generalized body pain and joint pain, associated with right knee joint swelling for the past three days with no history of fever or trauma. He gave a history of intermittent abdominal pain, which was diffuse and not associated with vomiting or loose stools. He did not have melena. He had a varicella infection, about three months ago, which got resolved, not requiring the need for hospitalization.

His vital parameters, on arrival were as follows: blood pressure (BP) 150/90 mmhg, pulse rate (PR) 90/min, respiration rate (RR) 14/min, and temperature 98 F. General examination revealed trace edema at the ankles and arthritis involving the right knee, shoulder, and both elbows. There was minimal effusion in the right knee joint. Examination of the abdomen revealed mild tenderness in all quadrants of the abdomen.

**Figure 1:**
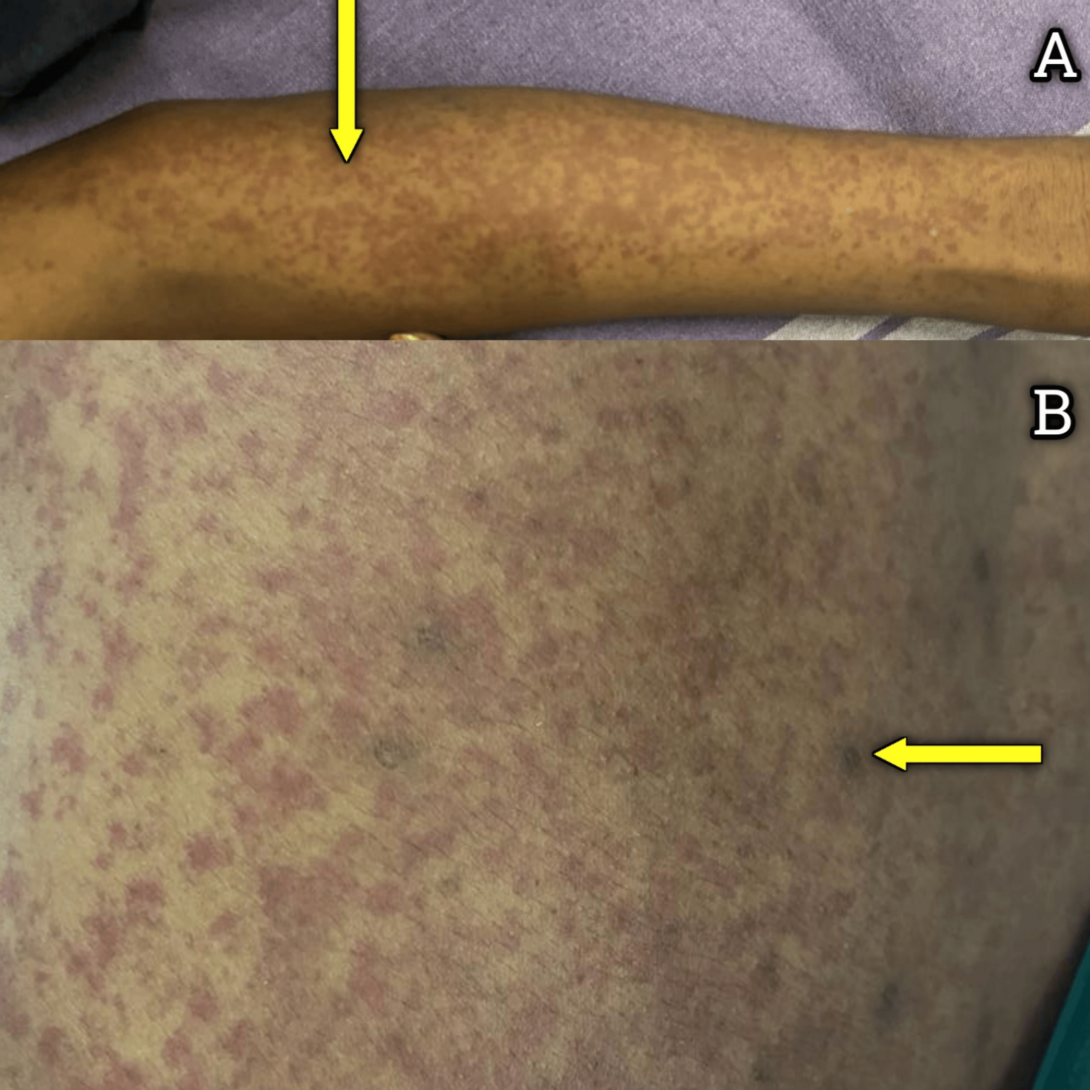
(A) Image depicts erythematous, palpable purpura observed in the upper limb (yellow arrow). (B) Image depicts erythematous, palpable purpura over the back (yellow arrow).

On the next day, he developed generalized multiple erythematous, palpable purpura, which were non-pruritic and non-blanching in nature with highly colored urine. Figure 1A-1B depicts the erythematous palpable purpura.

The lab parameters are mentioned in Table 1.

**Table 1:** Laboratory investigation results of our patient. Sub-nephrotic range proteinuria with hematuria and normal complement levels were observed. RBS: random blood sugar, urine ACR (spot): urine albumin-creatinine ratio, ANCA: anti-neutrophilic cytoplasmic antibody, hpf: high-power field

Parameters	Results	Reference
Blood		
Hemoglobin (g/dl)	13.9	13-17
White blood cells (cells/cubic.mm)	12,460 (N-87.8%, L-8.8%)	4000-11000
Neutrophils (%)	87.8	40-80
Lymphocytes (%)	8.8	20-40
Platelet count (cells/cubic.mm)	2,78,000	1,50,000-4,10,100
Erythrocyte sedimentation rate (mm/hr)	35	0-10
Prothrombin time/international normalized ratio	17.5/1.27	Control:14
Activated partial thromboplastin time	25.7	Control:31
RBS (milligram/deciliter)	124	<200
Urea (milligram/deciliter)	27	17-43
BUN (milligram/deciliter)	13	6-20
Creatinine (milligram/deciliter)	0.8	0.7-1.3
Urine analysis		
Appearance	Turbid	
Albumin	2+	
Red blood cells (/hpf)	Plenty	<5/hpf
White blood cells (/hpf)	10-12/hpf	<5/hpf
Epithelial cells	2-3/hpf	
Cast/crystals	Not found	
Urine ACR (Spot)(mg/g)	336	<30
24-h urine protein (g)	0.78	<0.15
Immunoassay		
Anti-nuclear antibody	Negative	
ANCA	Negative	
Complement C3 (milligram/deciliter)	94	75-175
Complement C4 (milligram/deciliter)	26	15-45

**Figure 2:**
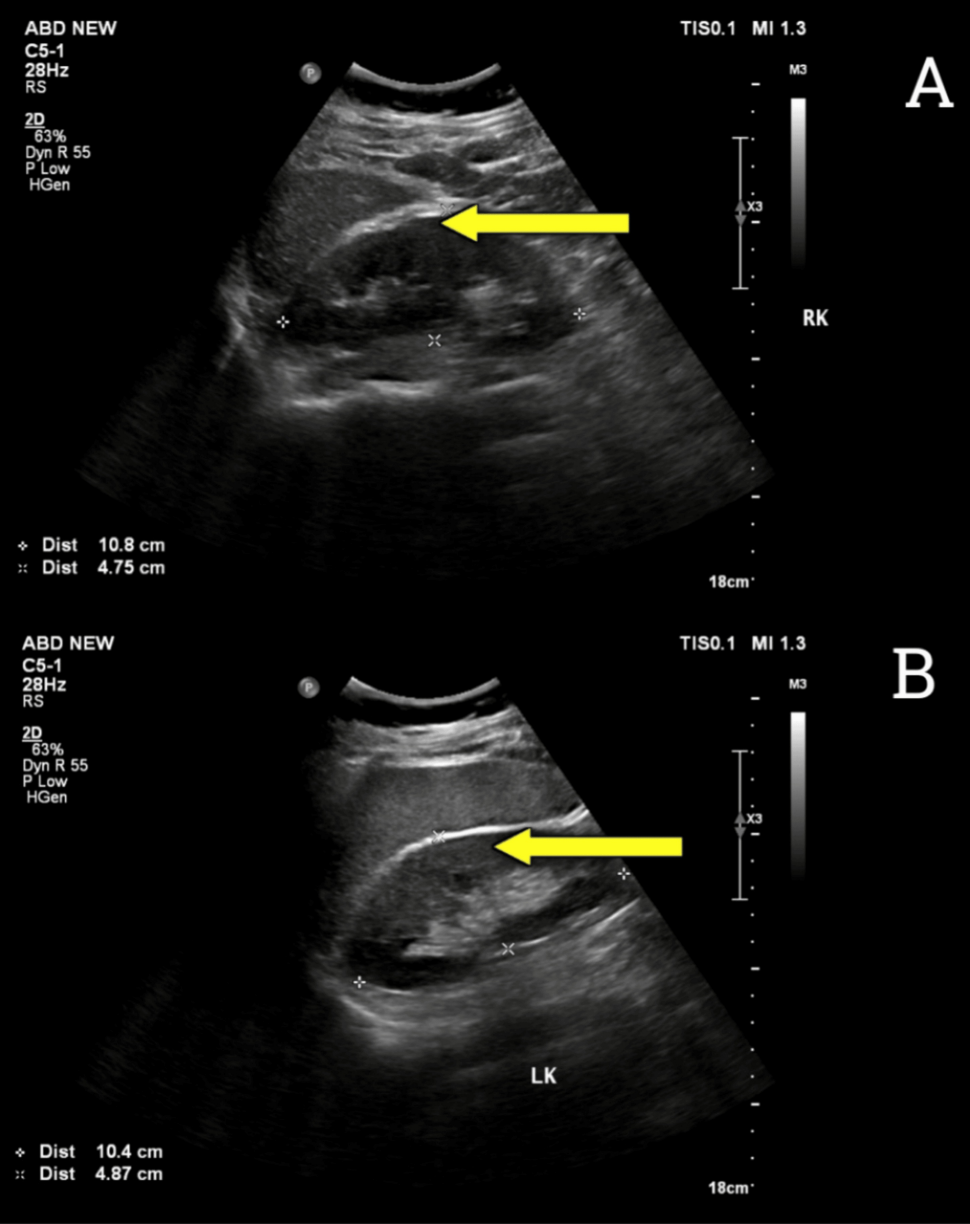
(A) Ultrasonography of the right kidney (yellow arrow), measuring 10.8 cm x 4.75 cm with normal renal parenchymal echoes and maintained corticomedullary differentiation with no pelvicalyceal system dilation. (B) Ultrasonography of the left kidney (yellow arrow), measuring 10.4 cm x 4.87 cm with normal renal parenchymal echoes and maintained corticomedullary differentiation, with no pelvicalyceal system dilation. cm: centimeter

Ultrasound abdomen showed no significant abnormality in the kidneys and urinary tract (Figure 2).

A diagnosis of acute nephritic syndrome was made, considering the clinical presentation and corroborative evidence from blood and urine investigations.

**Figure 3:**
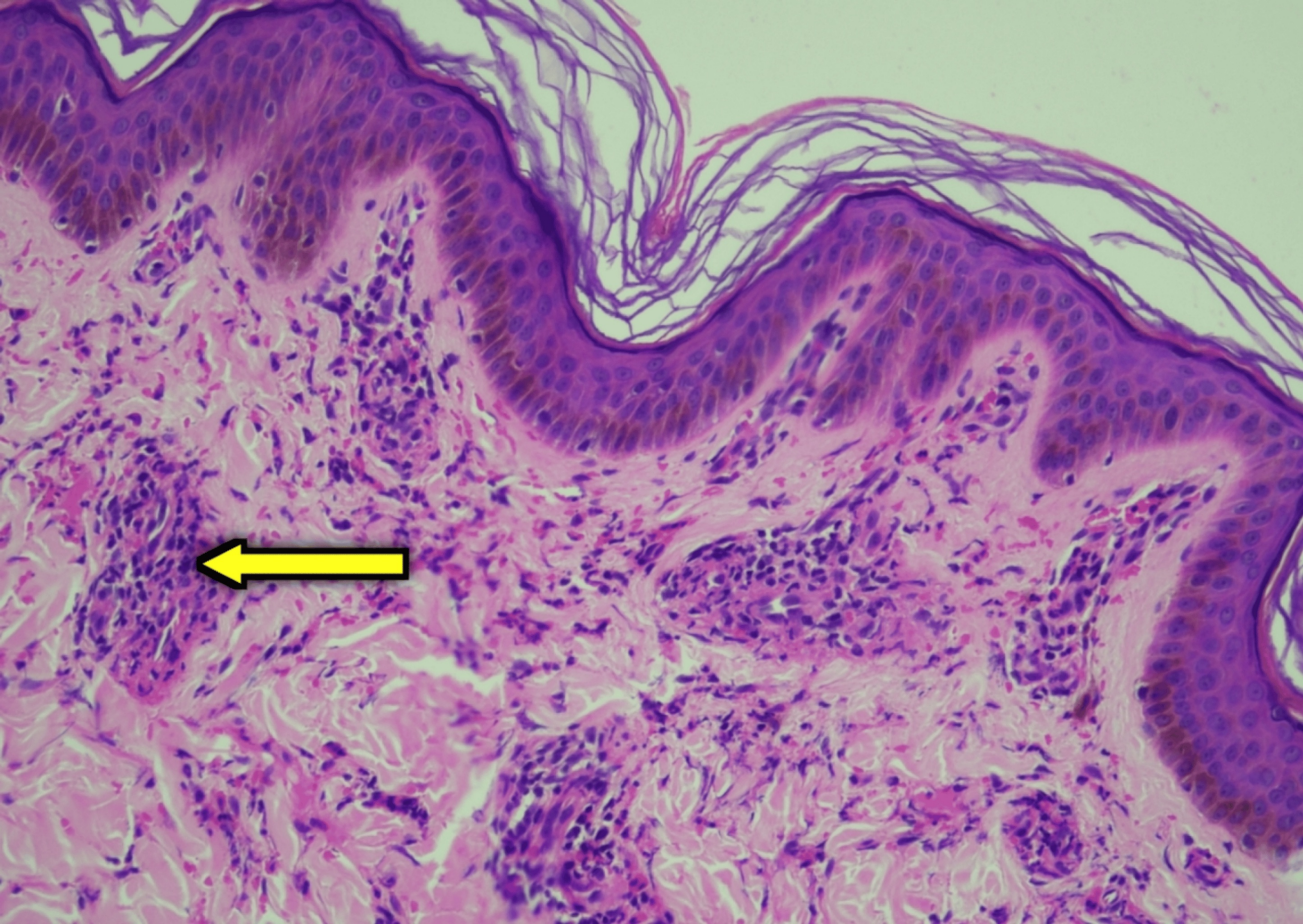
Skin biopsy from left forearm shows perivascular and endovascular neutrophilic aggregates(yellow arrow), nuclear debris, occasional eosinophils, and fibrin deposits.

Possible causes for nephritic syndrome were analyzed. ANA and ANCA were found to be negative. Anti-streptolysin O (ASO) titers and C3 and C4 levels were done and found to be within normal limits. A skin biopsy was performed on the purpuric lesions on the left forearm, which revealed leucocytoclastic vasculitis (Figure 3).

**Figure 4:**
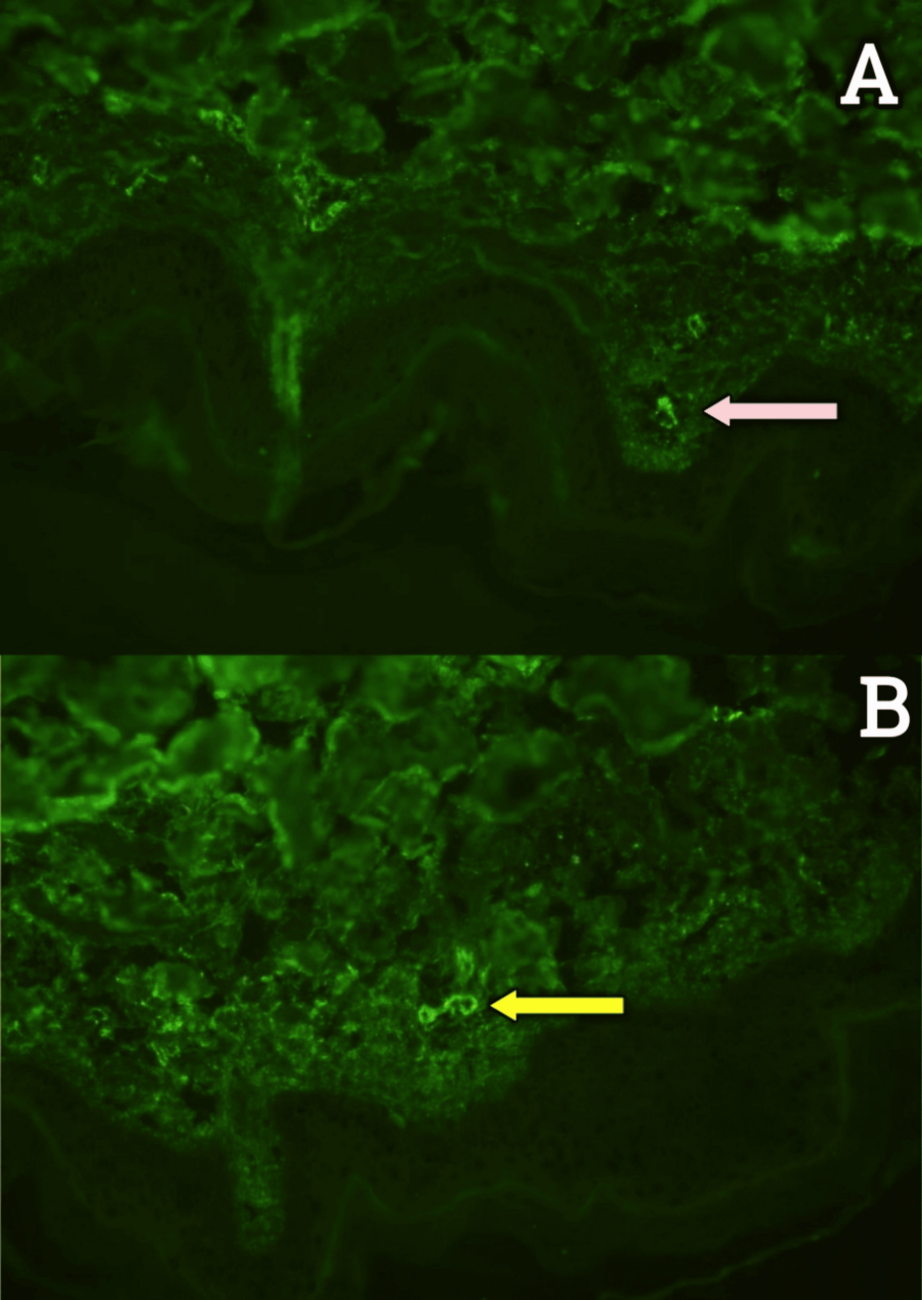
(A) Granular deposits of C3 (white arrow) seen in the papillary dermis under immunofluorescence staining. (B) Granular deposits of IgA (yellow arrow) seen in the papillary dermis under immunofluorescence staining. C3: complement C3; IgA: immunoglobulin A

Figure 4A-4B depicts the immunofluorescence image showing IgA and C3c deposits.

**Figure 5:**
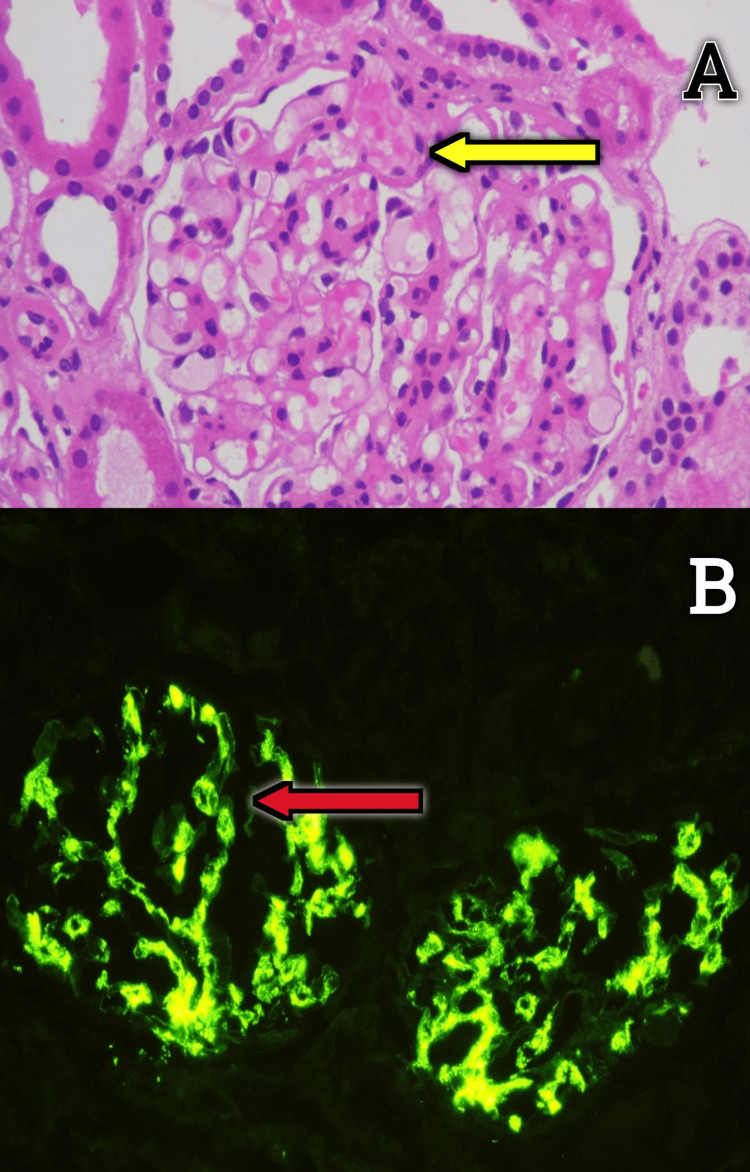
(A) Kidney biopsy: light microscopy (200x magnification) showing mesangial hypercellularity (yellow arrow) under hematoxylin and eosin stain. (B) Kidney biopsy: Diffuse granular IgA deposits in the mesangium (red arrow) under immunofluorescence staining. IgA: immunoglobulin A

A diagnosis of IgA vasculitis was made, and he was started on steroids (prednisolone), initially at a dose of 1 mg/kg/day for two weeks followed by 0.5 mg/kg/day for the subsequent two weeks. A kidney biopsy was done, which revealed mesangial hypercellularity with mesangial deposits of IgA and C3, with no crescents, suggestive of IgA vasculitis nephritis (Figure 5A-5B).

He also received a short course of angiotensin receptor blocker (telmisartan 40 mg OD). He made a full recovery over a period of one month. Corticosteroids were tapered over a month, and no relapse has been observed since then.

## Discussion

IgAV, an immune-mediated vasculitis, is the most common form of vasculitis in children, but rare in adults [6]. IgAV can involve various organ systems, such as the skin, gastrointestinal tract, and musculoskeletal system. When the kidney is involved, it is referred to as IgA vasculitis nephritis (IgAVN). It can rarely involve the central nervous system (cerebral vasculitis, presenting as cerebral hemorrhage) and respiratory system (presenting as pulmonary hemorrhage) [7].

EULAR classification criteria for IgAV are as follows: mandatory criteria (non-thrombocytopenic palpable purpura, predominantly in the lower limbs plus one of the four criteria, viz., renal manifestations, abdominal pain, arthralgia/arthritis, and leukocytoclastic vasculitis with IgA deposits in the skin biopsy).

Kidney involvement in IgAV in children is about 20-54%, whereas in adults, it is more common (up to 80%) and usually more severe [8]. Macroscopic hematuria is the presenting feature in 40-50% of patients with IgAN, with asymptomatic microscopic hematuria and proteinuria being present in 30-40% of patients [9]. The risk of progression to chronic kidney disease is common in adults, with a recent paper suggesting that 50% of patients reach kidney failure or die in a median follow-up of 5.9 years. The risk is higher in patients with greater time-averaged proteinuria [10]. The most common renal biopsy lesions are focal or diffuse mesangial proliferative glomerulonephritis; however, a variety of lesions have been described, including crescentic glomerulonephritis [11].

Pathogenesis of this disease is based on an interplay of various genetic and environmental factors. Several infections, vaccines, drugs, and food components can trigger the onset of disease in genetically vulnerable individuals. The infectious triggers include Group A *Streptococci*, *Mycoplasma pneumoniae*, *Helicobacter pylori*, *Campylobacter*, *Yersinia*, *Shigella*, *Salmonella*, *Brucella*, *Legionella*, adenovirus, SARS-CoV-2, parvovirus, respiratory syncytial virus (RSV), parainfluenza virus, and varicella zoster virus (VZV) [12,13]. Certain variants of genes encoding angiotensin-converting enzyme, endothelial nitric oxide synthase, and interleukin -18 have been identified as conferring vulnerability to the disease [14].

A four-hit hypothesis has been put forth to describe the pathogenesis of IgAV. One widely accepted theory details the IgA1 lacking the galactose residues (Gd-IgA1), associated with aberrant glycosylation and the subsequent development of antibodies (IgG and IgA) against this immunoglobulin residue (Gd-IgA1). This further leads to the formation of immune complexes, consisting of antibodies IgA and IgG in combination with Gd-IgA1 residue, which gets deposited in various sites such as the skin (endothelium), kidneys, gastrointestinal tract, and musculoskeletal system (joints) [15].

In our patient, there was no immediate trigger that could be identified. However, since the patient had a varicella infection three months before presentation, it is possible that this may have contributed to the development of IgAV, although it cannot be confirmed. However, the few case reports describing the association between IgAV and varicella infections describe a much shorter latent period.

Another hypothesis invokes cross-reactive antibodies generated against the pathogens, affecting the vascular endothelial cells of various organ systems (IgA1-AECA) [16]. Immune complexes activate alternate complement pathways and IgA can activate the lectin pathway implicating a pathogenic role of complement. However, an interesting point to note is that the antibodies generated against the abnormal IgA, belonging to the IgG class, surprisingly are not detected in biopsies from the skin or the kidney, emphasizing on need for further research to explore the pathogenesis of this condition [17].

Being a self-resolving disease, IgAV in children is treated with supportive measures only. The role of steroids in IgAVN is controversial. It is recommended for patients with a high risk of progressive CKD despite maximal supportive care (defined as proteinuria >1 g/day despite three months of optimized supportive care) and for patients with rapidly progressive IgAN (defined as >50% decline in eGFR of <3 months) [18]. For cutaneous manifestations of IgAV, some report that it may be required in painful or ulcerative disease and that it may help prevent new lesion formation [19]. In addition, steroids may have some role in the management of arthralgias and abdominal symptoms in severe or refractory cases [20]. In our patient, in view of the florid clinical presentation with nephritis, arthralgia, abdominal pain, and diffuse leukocytoclastic vasculitis, it was decided to administer a trial of steroids. 

## Conclusions

This case describes the classic presentation of IgAV in an adult with arthritis, gastrointestinal symptoms, leukocytoclastic vasculitis, and IgA nephropathy. Despite IgAV typically being a disease of childhood, patients do present even in adulthood. While the precise role of steroids in the management of these patients is unclear, our patient had a good response to a short course of low-dose steroids. Patients with IgAV are typically excluded from most trials of IgA nephropathy, and thus treatment recommendations for IgA nephropathy are usually extrapolated to IgA vasculitis. More research focussing specifically on therapies for IgAV is warranted in the future.
